# The Signaling Pathway That cGAMP Riboswitches Found: Analysis and Application of Riboswitches to Study cGAMP Signaling in *Geobacter sulfurreducens*

**DOI:** 10.3390/ijms23031183

**Published:** 2022-01-21

**Authors:** Zhesen Tan, Chi Ho Chan, Michael Maleska, Bryan Banuelos Jara, Brian K. Lohman, Nathan J. Ricks, Daniel R. Bond, Ming C. Hammond

**Affiliations:** 1 Department of Chemistry and Center for Cell & Genome Science, University of Utah, Salt Lake City, UT 84112, USA; zhesen.tan@utah.edu (Z.T.); bryan.banuelos@utah.edu (B.B.J.); nathan.ricks@utah.edu (N.J.R.); 2 Department of Plant and Microbial Biology and BioTechnology Institute, University of Minnesota, Minneapolis, MN 55455, USA; cchan@umn.edu (C.H.C.); males017@umn.edu (M.M.); 3 Huntsman Cancer Institute, University of Utah, Salt Lake City, UT 84112, USA; brian.lohman@hci.utah.edu

**Keywords:** riboswitch, reporter, biosensor, cyclic dinucleotide, bacterial signaling

## Abstract

The Hypr cGAMP signaling pathway was discovered via the function of the riboswitch. In this study, we show the development of a method for affinity capture followed by sequencing to identify non-coding RNA regions that bind nucleotide signals such as cGAMP. The RNAseq of affinity-captured cGAMP riboswitches from the *Geobacter sulfurreducens* transcriptome highlights general challenges that remain for this technique. Furthermore, by applying riboswitch reporters in vivo, we identify new growth conditions and transposon mutations that affect cGAMP levels in *G. sulfurreducens*. This work reveals an extensive regulatory network and supports a second functional cGAMP synthase gene in *G. sulfurreducens*. The activity of the second synthase was validated using riboswitch-based fluorescent biosensors, and is the first known example of an active enzyme with a variant GGDDF motif.

## 1. Introduction

Cyclic dinucleotides (CDNs) are second messengers in bacteria that control global physiology. Cyclic di-GMP was the first to be discovered and remains the most widespread CDN signal that controls cell motility, attachment, and biofilm formation [1]. In the past decade, cyclic di-AMP [2], cGAMP [3], and more recently other cyclic di- and tri-nucleotides have been discovered [4,5,6]. These CDNs and CTNs have roles in different signaling pathways for diverse processes including stress response, surface sensing, and immune sensing.

So far, natural riboswitches have been identified that regulate genes by binding to cyclic di-GMP, cyclic di-AMP, and cGAMP [7,8,9]. While the two riboswitch classes for cyclic di-GMP and cyclic di-AMP have distinct RNA folds [10,11], the cGAMP riboswitch is structurally related to the cyclic di-GMP riboswitch, with natural sequence variations in the binding pocket that enable the riboswitch to selectively recognize cGAMP [12]. Interestingly, bacteria of the *Geobacter* genus have a particularly high abundance of cGAMP riboswitches. However, we and others also have discovered two nonhomologous families of enzymes that make cGAMP, DncV/CD-NTases and Hypr GGDEFs, which are broadly distributed in bacteria [5,13,14]. Therefore, we hypothesize that there may be novel riboswitches that sense cGAMP in other bacteria.

*Geobacter sulfurreducens*, a model organism for the study of electron transfer to metals, must sense and attach to conductive surfaces that act as electron acceptors. In *Geobacter sulfurreducens*, knocking out the Hypr GGDEF enzyme GacA results in 2–16-fold reduction in transcripts carrying cGAMP riboswitches as analyzed by RNA-seq, which we showed ultimately affects extracellular electron transfer to Fe(III) oxides [15]. A riboswitch-luciferase reporter also permitted analysis of cGAMP levels in Δ*gacA* and strains complemented with different GacA mutants that affect enzyme activity. This led us to hypothesize that the riboswitch reporter could be used in a high-throughput screen for growth conditions and/or genes that affect cGAMP levels in *G. sulfurreducens*.

**Figure 1:**
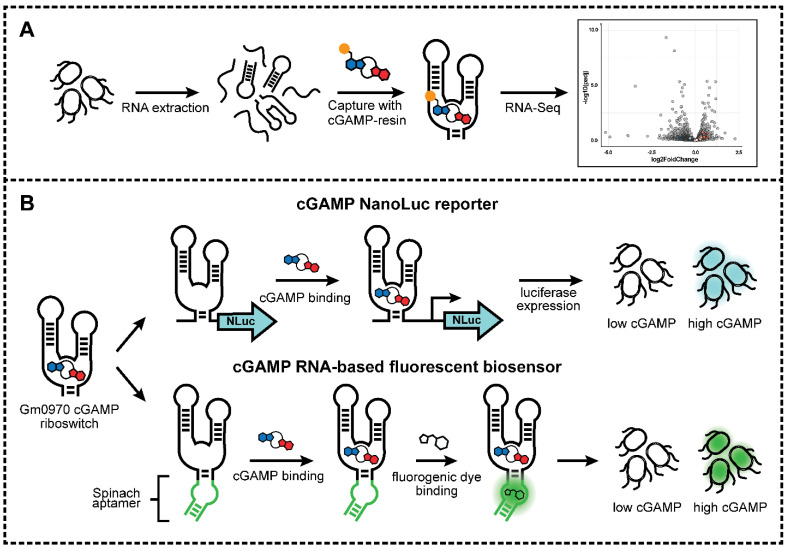
Overview of (**A**) riboswitch analysis and (**B**) riboswitch applications featured in this study.

In this collaborative study, we use the bacterium *Geobacter sulfurreducens* as a model for the analysis and application of riboswitches to study the cGAMP signaling pathway (Figure 1). We first test a method to identify mRNA transcripts containing cGAMP riboswitches by performing an affinity-based method on the *G. sulfurreducens* transcriptome. While initial in vitro experiments showed that a cGAMP riboswitch could be modestly enriched by affinity capture to cGAMP-Sepharose beads, RNAseq analysis of bound versus input mRNAs revealed that this enrichment was overridden by other factors when total RNA was evaluated. In contrast to the in vitro results, a riboswitch-luciferase reporter and a riboswitch-based fluorescent biosensor were successfully applied in several cell-based screening experiments. These results highlight a second cGAMP cyclase with a non-canonical GGDDF active site motif, induction of cGAMP by specific amino acids, and new genes that increase or decrease cGAMP levels in vivo.

## 2. Results

### 2.1. Riboswitch Analysis: Development of cGAMP Riboswitch Affinity Capture Method

Previously, researchers have conjugated the cyclic dinucleotides, cyclic di-GMP and cyclic di-AMP, to Sepharose beads in order to perform proteomics experiments to identify protein effectors for these signaling molecules [16,17]. Thus, we considered whether a similar approach could be applied to perform transcriptomics experiments to identify novel riboswitches for cGAMP or other newly identified nucleotide signals. First, enzymatically synthesized and HPLC-purified cGAMP was covalently conjugated to epoxide-activated Sepharose beads. Successful conjugation could be observed by the Sepharose beads gaining absorbance at 260 nm (Figure S1A). Any remaining unreacted epoxide groups were quenched by reacting with ethanolamine. Blank Sepharose beads were prepared as a control by performing the same procedure without cGAMP.

**Figure 2:**
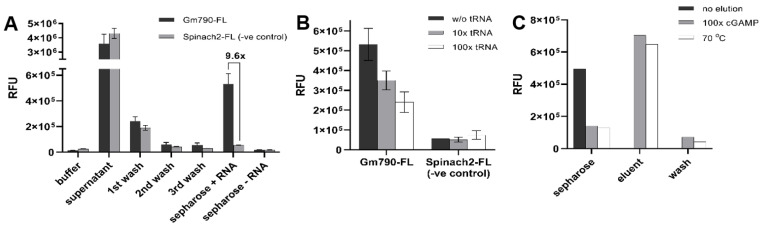
In vitro analysis of fluorescently labeled Gm790 riboswitch aptamer binding to cGAMP-Sepharose column. (**A**) Retention of Gm790 riboswitch compared to a control RNA during different affinity capture steps. (**B**) Effect of excess non-specific RNA (yeast tRNA) on RNA retention. (**C**) Analysis of different elution methods.

To test the ability of riboswitches to bind specifically to cGAMP-Sepharose, the riboswitch aptamer called Gm0970 was 3′-end labeled with fluorescein (Figure S1B). This riboswitch aptamer from *Geobacter metallireducens* is ~1600-fold selective for cGAMP over cyclic di-GMP and has a dissociation constant (*K*_D_) of ~16 nM for cGAMP [8]. As a control structured RNA, the Spinach2 aptamer was also fluorescently labeled. Binding experiments were performed by incubating the RNAs with cGAMP-Sepharose or blank Sepharose, followed by three washes. Aliquots of the supernatant, washes, and beads were analyzed for the presence of fluorescently labeled RNAs (Figure 2A). The data show that Gm0970 is retained 9.6-fold over the control RNA on cGAMP-Sepharose. In contrast, little to no fluorescence signal was observed for both RNAs with blank Sepharose. The binding experiments were also performed with or without excess yeast tRNA to examine the effect of non-specific RNAs on the retention of specific and control RNAs (Figure 2B). Some loss of specific retention (1.5× and 2.0×) was observed when beads were incubated with Gm0970 in a mixture with a high excess of tRNA (10× and 100×). Together, these results show that the riboswitch aptamer maintains specific binding to Sepharose-conjugated cGAMP.

We next tested two different elution methods, competitive elution with 5 mM cGAMP or heat (70 °C), for efficient collection of cGAMP-bound RNAs. Aliquots of the Gm0970-bound beads before and after treatment, the eluent, and post-elution wash were analyzed by fluorescence (Figure 2C). The data show that both methods were comparably effective, so the competitive cGAMP was adopted moving forward to provide higher specificity to the elution step.

### 2.2. Riboswitch Analysis: Transcriptome Affinity Capture and RNA-Seq Analysis

Having demonstrated selective binding and elution of the Gm0970 aptamer sequence with cGAMP-Sepharose, we next performed affinity capture and RNA-seq analysis on the *Geobacter sulfurreducens* transcriptome. Our prior bioinformatics analysis of the *G. sulfurreducens* genome revealed that there are 17 GEMM-I class riboswitches, of which 12 were shown experimentally to bind cGAMP. One of these riboswitch aptamers, Gs1761, has a very tight affinity (*K*_D_ of ~530 pM) for cGAMP and has been structurally characterized by *x*-ray crystallography. Furthermore, we showed that levels of these endogenous cGAMP riboswitch-regulated transcripts were decreased (2- to 16-fold) when the GMP-AMP cyclase GacA was knocked out. Thus, the *G. sulfurreducens* transcriptome serves as an important proof-of-principle case where the locations of bona fide cGAMP riboswitches are known.

To obtain mRNA, *G. sulfurreducens* was harvested in the mid-exponential phase during growth, with fumarate as the electron acceptor. Total RNA was isolated from cell lysate and analyzed on an Agilent TapeStation, which showed an average RNA Integrity Number (RIN) value of 9.0 ± 0.1, indicating the high quality of the isolated RNA. After performing an rRNA depletion step, the samples of total transcripts from *G. sulfurreducens* were pooled, renatured, and then incubated with either cGAMP-Sepharose or blank Sepharose followed by elution with cGAMP. An aliquot of total transcripts was saved as the input. The experiment was performed with two different concentrations of total transcript RNAs (75 or 150 nM) used as input, and each RNA sample (input, cGAMP, or blank) was submitted for RNA-seq analysis.

Since RNAs were not fragmented prior to the affinity capture step, it was expected that full transcripts containing cGAMP-binding riboswitches would be enriched, rather than just the riboswitch region. Thus, bioinformatics analysis used the DESeq2 workflow and statistical methods commonly applied to differential gene expression [18]. Differential expression comparisons were performed between cGAMP versus blank, cGAMP versus input, and blank versus input, where differences in “expression” in fact are due to selective retention of transcripts on beads versus input.

**Figure 3:**
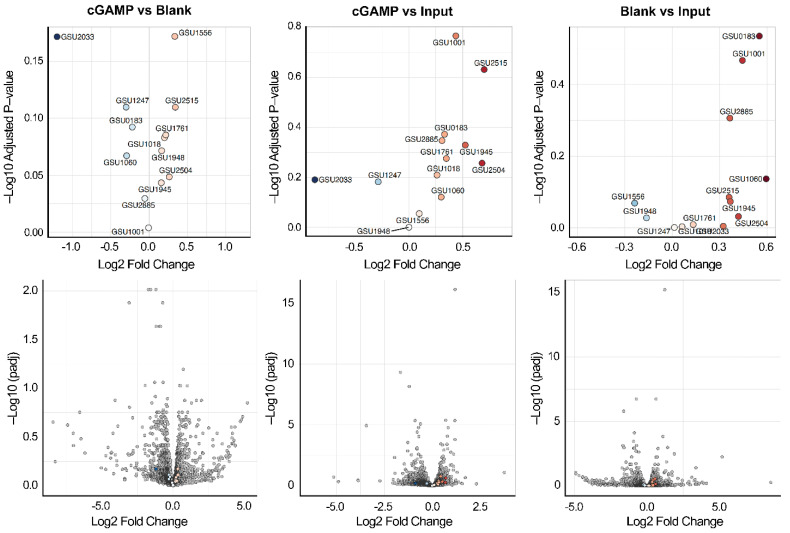
Volcano plots for DEseq analysis comparing cGAMP vs. blank (left panels), cGAMP vs. input (middle panels), or blank vs. input (right panels). Statistical significance (false discovery rate-adjusted *p*-values) versus magnitude of change (log2FoldChange) is shown. Top panels show only riboswitch genes, whereas bottom panels show riboswitch genes as colored points and all other bacterial genes as grey points. Riboswitch genes are colored relative to the 0 point on the x-axis: red points have a positive log2FoldChange and blue points have a negative log2FoldChange. Points become darker as the absolute value of the log2FoldChange increases.

There are 13 total GEMM-Ib riboswitches previously identified in *G. sulfurreducens* by bioinformatics and characterized for cGAMP binding activity [8]. All of the riboswitch-containing transcripts were present in the input samples, with two transcripts (GSU1761 and GSU1945) ranked among the top 5% of expressed genes (Table S1). Our expectation was that mRNAs containing these riboswitches would be significantly enriched either in the comparison between cGAMP versus blank or cGAMP versus input, whereas they would not be enriched in blank versus input. However, we found that none of the cGAMP riboswitch transcripts were significantly enriched in any of the three comparisons (Figure 3, Table S1). The false discovery rate-adjusted *p*-values (p-adj) for changes in transcript abundance in the treatments were above 0.05, meaning that any observed changes were not statistically significant. Furthermore, we saw the same trend of enrichment of riboswitch transcripts for both treatments (cGAMP or blank beads) over the input control, which indicates non-specific enrichment, and none of the riboswitch-related transcripts were most enriched in the treatment pools.

### 2.3. Riboswitch Application: Analysis of a Second Hypr GGDEF in Geobacter sulfurreducens

The above experiments show some of the challenges faced with in vitro analysis of the RNA transcriptome to capture riboswitch–ligand interactions. In contrast, we have found much better success to analyze riboswitches in vivo through application of riboswitch-based reporters and biosensors. Previously, the Gs1761 riboswitch was used to construct a cGAMP-activated nanoluciferase (Nanoluc) reporter that was integrated into the chromosome of *G. sulfurreducens*. Nanoluc activity decreased by 80% in the *gacA* deletion strain, consistent with biochemical data showing that GacA is a hybrid-promiscuous (Hypr) GGDEF enzyme that makes cGAMP, e.g., has cGAMP cyclase activity (GAC) [15].

**Figure 4:**
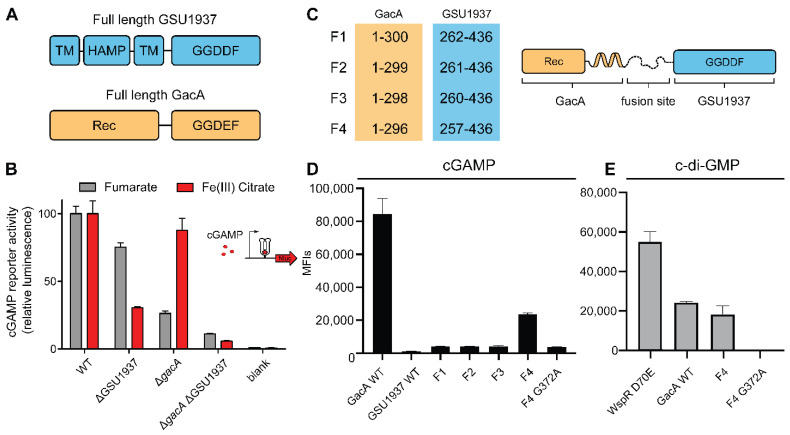
Demonstration that GSU1937, an enzyme with a non-canonical GGDDF motif, is a catalytically active signaling enzyme. (**A**) Protein domain architecture of GSU1937 and GacA enzymes with the sequence of the catalytic motif. (**B**) Activity of the cGAMP riboswitch luciferase reporter in *G. sulfurreducens.* In the absence of intracellular cGAMP, incomplete transcription of the Gs1761 riboswitch prevents the production of downstream mRNA encoding nanoluciferase. Deletion of GSU1937 reduced cGAMP reporter activity in both WT and strains lacking the known cGAMP synthase GacA, indicating that GSU1937 is also a cGAMP cyclase. (**C**) Fusion sites for constructs made to test the activity of the GGDDF catalytic domain. (**D**) Activity of riboswitch-based fluorescent biosensor for cGAMP in *E. coli* with co-expression of different enzyme constructs. GacA is a known cGAMP synthase. (**E**) Activity of riboswitch-based fluorescent biosensor for c-di-GMP in *E. coli* with co-expression of different enzyme constructs. WspR D70E is a known c-di-GMP synthase.

During preliminary experiments for a high-throughput screen in *G. sulfurreducens*, unexplained variability in reporter activity was observed. We hypothesized that this additional Nanoluc activity could be due to activity of a second cGAMP cyclase. GacA has a serine in the active site (Ser348) that hydrogen bonds with the Watson–Crick–Franklin face of either guanine or adenine nucleobases, which is critical to its acceptance of GTP and ATP as substrates to form cGAMP [13,15]. Of 28 other GGDEF-domain containing enzymes encoded in *G. sulfurreducens*, only GSU1937 also contains a Hypr-defining serine residue in the active site. Tests of GSU1937 with the in vivo biosensor screen in *E. coli* that identified GacA were inconclusive, potentially due to transmembrane HAMP domains that affected expression, activity, or localization in *E. coli* [13]. However, when GSU1937 was deleted from the *G. sulfurreducens* cGAMP reporter strain, Nanoluc activity under standard fumarate conditions decreased by ~25% (Figure 4A,B). A double *gacA GSU1937* deletion strain decreased total activity by over 90%, further suggesting that GSU1937 contributes to cGAMP production. When cells were grown with the electron acceptor Fe(III) citrate, deletion of GSU1937 had a larger impact on cGAMP synthesis, reducing reporter levels by 70%, while *gacA* deletion only reduced levels by 10%. This was the opposite of results from fumarate-grown cells, indicating that GSU1937 could be more important than GacA in maintaining cGAMP levels during the reduction of environmental metals such as Fe(III) citrate.

While GSU1937 contains the diagnostic Ser348 residue, it also has a modified GGDDF motif. It is known that GGEEF domains (such as *Caulobacter crescentus* PleD) can produce cyclic di-GMP [19], consistent with the broader active site motif definition of [G/A/S]G[D/E]E[F/Y], but other degenerate motifs are typically inactive and may act as binding domains to facilitate the activity of another domain. For example, the *Pseudomonas aeruginosa* PAO1 GGDDF/EAL-containing protein PA3258 fails to increase cyclic di-GMP levels or cyclic di-GMP-dependent phenotypes when overexpressed or deleted in *P. aeruginosa* PAO1 (4), and the GEDEF-EAL *Caulobacter crescentus* protein CC3396 only has phosphodiesterase activity [20].

Interestingly, in the case of GSU1937 and its many homologues within Deltaproteobacteria, no other catalytic domain (EAL or HD-GYP, which have cyclic dinucleotide phosphodiesterase activity) is present. Previously, we found that the fourth residue in the GGDEF motif is conserved as glutamate (E) because it acts in a cross-dimer fashion as the catalytic base that activates the substrate 2′-hydroxyl to perform the cyclization reaction [15]. The variation to aspartate (D) maintains side chain functionality, suggesting it could preserve catalytic activity, but on the other hand, the side chain being shorter could disrupt its cross-dimer function.

To further test and validate the activity of GSU1937, we decided to revisit the in vivo biosensor screen in *E. coli*. The engineered strain BL21 Star has no cGAMP activity, but does have basal c-di-GMP activity through many signaling enzymes. Reasoning that the issue was with the N-terminal transmembrane domain, a series of fusion proteins were made in which the active Hypr GGDEF domain of GacA was replaced with the Hypr GGDDF domain of GSU1937. A flexible linker region found in both GacA and GSU1937 was chosen as the fusion site, and several constructs were made that moved the fusion site by a few residues (Figure 4C). We previously found that over-expression of these enzyme constructs in *E. coli* usually causes dimerization even in the absence of activating signals, enabling the activity of these signaling enzymes to be assessed in vivo. By co-expressing enzyme constructs with riboswitch-based biosensors that are selective for cGAMP or c-di-GMP, synthase activity for each of these cyclic dinucleotides causes increase in cellular fluorescence that can be quantitated by flow cytometry.

While WT and three fusion constructs of GSU1937 exhibited no activity, F4 showed some cGAMP synthase activity (Figure 4D). This functional fusion appears less active than GacA WT, which is in line with its contribution to reporter activity in *G. sulfurreducens*, but also may be attributed to F4 being an artificial construct. To validate that the observed activity was due to a functional GGDDF domain, the G372A mutant with a GADDF motif was constructed and showed no activity as expected. We also tested F4 and G372A F4 fusions for c-di-GMP synthase activity compared to the original Hypr GGDEF, GacA (Figure 4E). The F4 fusion with the non-canonical GGDDF motif showed comparable c-di-GMP synthase activity to GacA, whereas the G372A F4 fusion with GADDF motif was inactive as expected.

### 2.4. Riboswitch Application: Screening for New Conditions and Genes That Affect cGAMP Levels in Geobacter sulfurreducens

**Figure 5:**
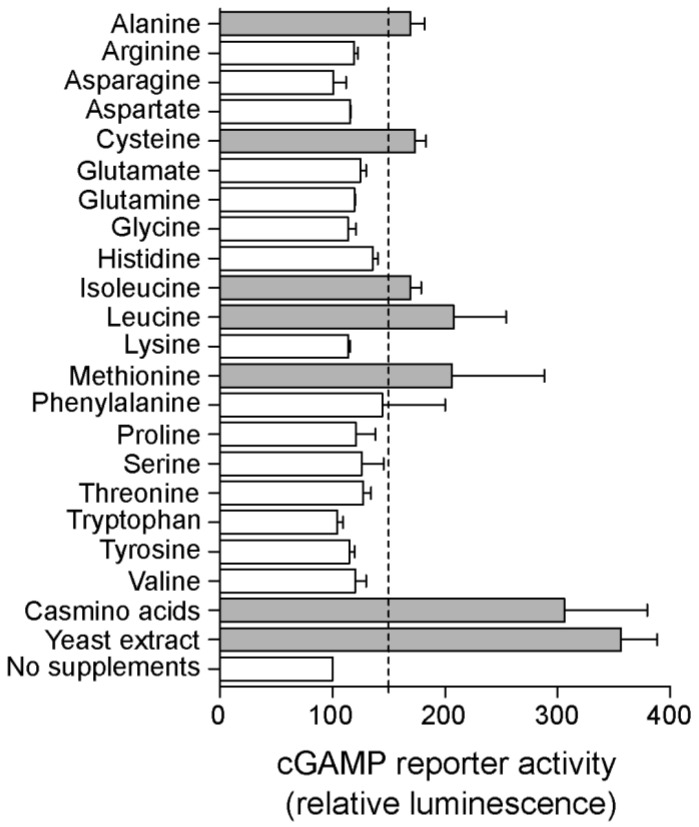
Specific supplements increase intracellular cGAMP levels. Both casamino acids and yeast extract increase activity of the cGAMP reporter in *G. sulfurreducens*. When single amino acids were added to minimal growth medium with acetate as the donor and fumarate as the acceptor, Leucine and Methionine stimulated cGAMP reporter activity the most, with Isoleucine, Cysteine, and Alanine also producing weak effects. Conditions that give 150% or more reporter activity are highlighted as grey bars.

Previous LC-MS analysis showed that supplementing *G. sulfurreducens* medium with yeast extract enhanced cGAMP levels in cell extracts [8]. When yeast extract was added to the medium of the cGAMP reporter strain, Nanoluc activity increased ~3 fold compared to minimal medium-grown cells, indicating there are compounds in yeast extract that elevate intracellular cGAMP levels (Figure 5). Further screening found that this dramatic increase in activity also occurred when the cGAMP reporter strain was supplemented with casamino acids, consistent with at least one of the signals being an amino acid. Out of 20 individual amino acids added at sub-nutritional concentrations, alanine, cysteine, isoleucine, leucine and methionine each increased Nanoluc activity more than 1.5 fold (Figure 5), showing that intracellular cGAMP levels can be induced by specific amino acids.

The presence of a receiver (Rec) domain on GacA and the finding that amino acid inputs increase cGAMP levels suggested additional regulatory pathways remained within the 92 sensor histidine kinases and 33 methyl-accepting chemotaxis proteins encoded in the *G. sulfurreducens* genome [21]. We constructed a non-saturating transposon library in the cGAMP reporter strain and screened the library for altered nanoluc activity to determine if an unbiased approach could discover new mutations that affected cGAMP levels. Colonies were arrayed onto 96-well plates that also included the cGAMP reporter in three backgrounds to act as controls for calibration: WT, *gacA* deletion (~20% of WT activity) and *esnD* deletion (~150% of WT activity). Each plate was manually assayed for reporter luciferase activity. Transposon mutants with <~50% and >~150% of WT nanoluc activity were re-isolated, re-tested and sequenced to identify the transposon disruption that altered cGAMP levels.

Five insertions were identified that decreased cGAMP-nanoluc activity, and three that increased activity (Table 1). Several transposon mutants were located in Type VI pili (*pil*) and nearby exopolysaccharide (*xap*) operons, with multiple unique insertions in *pilB* and *xapK*. A disruption in *pilA-C,* a subunit of the Type VI pilus recently proposed to be involved in protein secretion [22], decreased cGAMP levels, and disruptions within the extracellular polysaccharide operon downstream of *pilA-C* also decreased cGAMP to similar levels. In contrast, a disruption in *pilB*, the motor protein responsible for pilus extension, increased cGAMP. This pattern suggests a role for cGAMP in communicating protein secretion, as unpolymerized pilins (which would accumulate in ∆*pilB*) stimulated cGAMP synthesis, while the absence of pilin or extracellular anchoring materials created the opposite effect [23,24].

Two disruptions likely related to the negative allosteric effect of cyclic di-GMP on GacA activity were also isolated. Previously, we showed that cyclic di-GMP binds to the inhibitory site (I-site) for Hypr GGDEF enzymes to reduce catalytic activity in vitro [25], which matches the regulatory logic for in vivo results showing that deletion of the diguanylate cyclase EsnD increases cGAMP levels. Disruption of *esnB*, encoding a CheW-family protein involved in signaling to EsnD (10)*,* increased cGAMP reporter activity as expected. A transposon insertion in an intergenic region between two hypothetical proteins did not immediately explain altered cGAMP reporter activity, but whole genome sequencing revealed an off-site 11 bp deletion in a GGDEF-EAL enzyme (GSU0946) that could also increase cyclic di-GMP levels and allosterically inhibit GacA [15]. These findings from an exploratory transposon library indicate that many aspects of cGAMP regulation intersect with pilus function and surface binding, as well as control of the cyclic di-GMP pool via a putative phosphodiesterase.

Since the unbiased screen did not identify new histidine kinases and methyl-accepting chemotaxis proteins to be involved in cGAMP signaling, we also performed a targeted bioinformatics analysis of Cache domain-containing genes. Cache domains are small molecule-binding domains commonly associated with signaling enzymes and chemotaxis receptors. Several Cache domains from methyl chemotaxis proteins have been found to bind amino acids, including ones with solved *x*-ray crystal structures such as PctA from *Pseudomonas aeruginosa* that binds most amino acids [26], Mlp24 from *Vibrio cholerae* that binds serine, alanine, and arginine [27], and Tlp3 from *Campylobacteri jejuni* that binds nonpolar amino acids [28].

Nine genes in the *G. sulfurreducens* genome are annotated to have Cache domains, including four histidine kinases, four methyl chemotaxis proteins, and one GGDEF enzyme. The structures of each of these Cache domains were de novo modeled using the Robetta server due to low homology to other Cache domains [29], then the ligand binding pockets were defined using the program fpocket [30]. The DeeplyTough algorithm then was used to compare the modeled Cache binding pockets to those for Cache domains with known ligands and structures (Table 2) [31]. Interestingly, the GGDEF enzyme as well as the two histidine kinases showed high binding pocket similarity to different Cache domains that bind amino acids or dicarboxylate metabolites, whereas the other Cache domains did not. Thus, bioinformatics analysis reveals these three genes (GSU2388, GSU2507, GSU3356) as promising leads for future studies to understand the signaling logic that leads to regulation of cGAMP levels by amino acids.

## 3. Materials and Methods

### 3.1. Preparation of cGAMP-Sepharose Capture Compound

To prepare the Sepharose for coupling, 50 mg of Sepharose (Cytiva, Marlborough, MA, USA), which swells into ~175 μL, was washed with 2 mL of H_2_O and repeated 5 times. Each wash was allowed to sit for 10 min. The H_2_O was removed after the final wash and the Sepharose was resuspended in 175 μL of coupling buffer (100 mM carbonate buffer at pH 9.5).

To carry out the coupling with cGAMP, 50 μL of Sepharose was transferred into a new tube and 15 μmol of cGAMP resuspended in 50 μL coupling buffer was added into the same tube. The mixture was then incubated in a shaker set to 350 rpm at 55 °C for 48 h of coupling time. Once the incubation was complete, the unreacted cGAMP in the solution was removed, leaving only cGAMP-Sepharose in the tube. cGAMP-Sepharose was washed twice with 1M ethanolamine at pH 8 to completely remove any remaining cGAMP in the mixture.

Then, the unreacted epoxy groups on cGAMP-Sepharose were quenched with 1M ethanolamine at pH 8 in a shaker set to 350 rpm at 40 °C for 12 h. After quenching, the excess ethanolamine was carefully removed using a pipette. Finally, cGAMP-Sepharose was washed with 3 cycles of alternating pH (0.1 M acetate buffer pH 4.0 containing 0.5 M NaCl followed by 0.1 M Tris-HCl buffer pH 8 containing 0.5 M NaCl). For storage, cGAMP-Sepharose was resuspended in equal volume of coupling buffer and kept at 4 °C.

### 3.2. RNA Labeling with Fluorescein

For experiments using Gm0970, to test its binding to cGAMP-Sepharose, both Gm0970 and Spinach2 (as a negative control) were labeled with fluorescein on the 3′-end to allow for fluorescence readout. First, 10 μL of RNA and 54 μL of NaIO4 with final concentrations of 5 μM and 2 mM, respectively, were added into a tube and incubated on ice for an hour. Then, the oxidized RNA was precipitated using ethanol and the RNA pellet was washed once with cold 70% ethanol. Excess ethanol was removed and further dried under nitrogen, and the dried RNA was resuspended in 50 μL of 100 mM NaOAc buffer for the next step.

Next, 0.64 μL of 100 mM fluorescein carbazide (Thermo Fisher Scientific, Waltham, MA, USA) was added to the RNA solution, with a final concentration of 1 mM, and incubated on ice for 16 h. Once the incubation was complete, the labeled RNA was precipitated using ethanol and the RNA pellet was washed once with cold 70% ethanol. Excess ethanol was removed and further dried under nitrogen and the dried RNA was kept at −20 °C for storage. To remove the impurities and excess dyes from the labeling step, both labeled Gm0970 and Spinach2 was gel purified on 6% denaturing PAGE and concentration was measured on Nanodrop.

To demonstrate the successful labeling of fluorescein, 10 μM of unlabeled and labeled Gm0970 were ran on 6% denaturing PAGE for 30 min under 140 V. The gel was stained with SYBR Gold (Thermo Fisher Scientific, Waltham, MA, USA) and imaged using a GE Typhoon Gel Imaging Scanner.

### 3.3. Binding of RNA Samples to cGAMP-Sepharose

For binding experiments using labeled Gm0970 and Spinach2, all samples were prepared in a binding buffer containing 10 mM HEPES, 125 mM KCl, and 10 mM MgCl_2_, pH 7.5.

First, 30 μL of 0.5 μM RNA in binding buffer was prepared in a tube and renatured on a thermocycler. Renatured RNA was then diluted to a final concentration of 0.2 μM and ~3 μL of cGAMP-Sepharose was added to the RNA solution. This mixture was incubated at 30 °C in a shaker with 350 rpm for 2 h to allow for the RNA to bind to cGAMP-Sepharose. Once the binding reaction was complete, the cGAMP-Sepharose was allowed to completely settle down in the bottom of the tube, and the supernatant containing unbound RNA was carefully removed using a pipette. Then, cGAMP-Sepharose was washed with 45 μL binding buffer three times to eliminate any remaining unbound RNA.

For cGAMP elution experiments, the cGAMP-Sepharose at this stage was treated with 75 μL of 5 mM cGAMP and the mixture was incubated at 30 °C for 1 h. For heat elution experiments, the cGAMP-Sepharose was resuspended in 75 μL of binding buffer and incubated at 70 °C for 5 min. Once the elution step was complete, the eluent was transferred into a new tube, and the cGAMP-Sepharose was washed once with 45 μL binding buffer. The initial supernatant and all the washes were collected for fluorescence measurements.

For fluorescence measurement, 40 μL of each solution was transferred into a 96-well plate and analyzed on a fluorescence plate reader. All the fluorescence data in this study were collected at 523 nm, unless specified otherwise.

For binding experiments using unlabeled *Geobacter* RNAs, 50 μL of rRNA-depleted *Geobacter* total RNA was prepared in binding buffer (10 mM HEPES, 125 mM KCl, 10 mM MgCl_2_, pH 7.5) at two concentrations (180 nM and 90 nM, respectively) and renatured using a thermocycler. Renatured RNA was added to ~10 μL of cGAMP-Sepharose, making the final RNA concentration ~ 150 nM and 75 nM for the two samples. In addition, the renatured RNA was added to ~10 μL of blank Sepharose for two control samples. The rest of the steps in the binding reaction, washing, and elution with cGAMP were the same as described above.

### 3.4. Total RNA Isolation and Quality Check

Total RNA was isolated from *Geobacter* cell pellets using the RNeasy Mini kit (Qiagen, Hilden, Germany). This protocol was adopted from the manufacturer and slightly modified to optimize its efficiency with *Geobacter sulfurreducens*. First, 3 mg of *Geobacter* cell pellet (wet cell weight, thawed on ice) was transferred into a 2.0 mL centrifuge tube, along with 80 mg of glass beads and 850 μL of RLT buffer. The mixture was placed on a TissueLyser II (Qiagen, Hilden, Germany) at 30 Hz for 8 min to fully lyse the cells. Once the cell lysis was complete, the resulting lysate was centrifuged at 10,000 rpm for 10 s, and the supernatant was transferred to a new 2.0 mL centrifuge tube containing 1 mL of 70% EtOH. The lysate mixture was transferred to a spin column (provided by the kit) and centrifuged at 10,600 rpm for 15 s to capture the RNA in the membrane. The RNA was then treated with Qiagen Rnase-Free DNase following the protocol provided (20 μL of DNase and 100 μL of RDD buffer). The column was then washed with 350 μL of RW1 buffer (10,000 rpm for 15 s), 500 μL of RPE buffer (10,000 rpm for 10 s), and 500 μL of RPE buffer (10,000 rpm for 2 min). Finally, 50 μL of Rnase free H_2_O was loaded to the column and centrifuged at 10,000 rpm for 30 s to elute the RNA into a collection tube.

For storage, flash freezing using liquid nitrogen was carried out for all total RNA samples, which were then kept in a −80 °C freezer until future use. All total RNA isolated from *Geobacter* were analyzed on TapeStation (Agilent Technologies, Santa Clara, CA, USA) to obtain the concentration and RIN value for quality check. To do so, RNA samples were prepared by diluting (100×) with Rnase-free H_2_O. A High Sensitivity RNA ScreenTape Analysis kit was used for the analysis and the exact steps provided in the manufacturer’s protocol were followed without any modifications.

### 3.5. rRNA Depletion

Before total RNA was used in any binding experiments, it was treated with a RiboMinus™ Transcriptome Isolation Kit (bacteria) (Thermo Fisher Scientific, Waltham, MA, USA) for ribosomal RNA depletion. All steps provided in the manufacturer’s protocol were followed without any modifications. Once rRNA depletion steps were completed, the dried RNA pellets were resuspended in 5.5 μL of Rnase-free H_2_O. rRNA-depleted samples were analyzed again on Agilent TapeStation to obtain the concentration. The High-Sensitivity RNA ScreenTape Analysis kit was used for the analysis and the exact steps provided in the manufacturer’s protocol were followed without any modifications.

### 3.6. RNAseq

RNAseq libraries were built with the NEB Next Ultra II Directional RNA Library Prep (New England Biolabs, Ipswich, MA, USA) with rRNA Depletion (bacteria). Libraries were sequenced on the NovaSeq 6000 (Illumina, San Diego, CA, USA) with the NovaSeq SP Reagent Kit for paired-end 50 bp reads. Optical duplicates were removed from raw reads with Clumpify (v38.34), adapters trimmed with CutAdapt (v2.8) and aligned to the *Geobacter sulfurreducens PCA* genome with BowTie2 (2.2.9). Genes were counted using FeatureCounts (1.6.3). Raw counts were filtered to remove genes that had fewer than 5 counts in all samples. Expression was modeled as a function of treatment (cGAMP/blank/input) using DESeq2 and following the standard DESeq2 workflow [18].

### 3.7. Growth and Transposon Library Generation

*G. sulfurreducens* strains were grown in anoxic basal medium with acetate (20 mM) as the electron donor and fumarate (40 mM) as the electron acceptor. Agar (1.5%) was added to the acetate-fumarate medium when culturing for clonal isolates under a H_2_:CO_2_:N_2_ (5:20:75) atmosphere in a vinyl anaerobic chamber (Coy) or an anaerobic workstation 500 (Don Whitley). The pH of the medium was adjusted to 6.8, buffered with 2 g/L NaHCO_3_, and purged with N_2_:CO_2_ gas (80:20) passed over a heated copper column to remove trace oxygen. All cultures were grown at 30 °C [32].

To produce a transposon library in the *G. sulfurreducens* cGAMP reporter strain [15], the kanamycin resistance cassette was removed from a strain which harbors a chromosomal integrated cGAMP-riboswitch (GSU1761 promoter) that activates a nLuc gene (Promega) in response to cGAMP. Mutagenesis was performed via conjugation as described in [33]. Briefly, 1 mL of *E. coli* transposon donor strain WM3064 carrying pMiniHimar RB1 grown overnight in LB supplemented with 0.3 mM of diaminopimelic acid and 50 µg/mL kanamycin was pelleted before combining with 1 mL of the recipient *G. sulfurreducens* cGAMP reporter strain [34]. The *G. sulfurreducens* and *E. coli* cell mixture was spotted on top of a 0.22 µm filter on an agar plate with acetate and fumarate inside the anaerobic chamber for 4 h before selecting for transposon integrants on agar plates with 200 µg/mL kanamycin.

Cells grown in one liter of anoxic medium were used for RNA extractions. WT *G. sulfurreducens* was grown to an OD 600 of 0.3 before pelleting with RNAprotect (Qiagen) and stored at −80 °C.

### 3.8. Deletion of GSU1937

A scarless gene deletion using a SacB–sucrose counter-selection strategy was used to delete GSU1937 from both WT and Δ*gacA* cGAMP reporter strains [32]. Briefly, flanking regions (~750 bp) of GSU1937 were amplified and combined using primer pairs GCTTACAAGCTTTGATGAGAACACCGTCGTACAGGC and GTAATTGCTTCCCGGCGTGCTGCTGGCGATCAACTTCTGGACG, CGTCCAGAAGTTGATCGCCAGCAGCACGCCGGGAAGCAATTAC and GCTTACTCTAGACGTTACCGGCAGCATACCTATTGAG, digested with HindIII and XbaI, and ligated into pK18mobsacB digested with the same restriction enzymes. The resulting plasmid was transformed into an *E. coli* donor strain and conjugated with *G. sulfurreducens* as described above.

### 3.9. Nanoluciferase (Nluc) Reporter Assay

*G. sulfurreducens* cGAMP reporter strains and cells from the transposon library were arrayed in 96-well plates, where each plate included triplicate wells of the parent WT cGAMP reporter strain, as well as cGAMP reporter mutant strains that had higher (Δ*esnD*) and lower (Δ*gacA*) cGAMP levels as controls [15], then grown for 48 h. Cells were lysed with Bugbuster (Millipore Sigma, Burlington, MA, USA) in a nanoglo reaction buffer and assayed according to the manufacturer’s protocol (Promega). Mutants with less than 50% or more than 150% of WT Nluc activity were re-isolated and retested. Genomic DNA from clonal isolates of candidates confirmed to have repeatable Nluc activity was extracted and sequenced using Illumina sequencing. Reads were mapped onto the reference genome specific to our lab strain [15] using Breseq [8] to identify transposon locations and other off-site mutations. To test the effect of various supplements on the cGAMP levels, the *G. sulfurreducens* cGAMP reporter strain was supplemented with 0.2% yeast extract, 0.2% casamino acids, and sub-nutritional concentration (0.5 mM) of each amino acid except for tyrosine (0.25 mM), grown in triplicate on 96-well plates, and Nluc activity was assayed using the same procedure as above.

### 3.10. Fluorescent Biosensor Assay

GacA-GSU1937 fusions and any mutant derivatives were cloned via PCR into the second promoter site of the pCOLA-Duet plasmid and sequence-confirmed through the DNA Sequencing Core Facility at the University of Utah. pCOLA-Duet-GacA, pCOLA-Duet-GSU1937 and pCOLA-Duet-WspR D70E were obtained from previously reported work. The pCOLA-Duet enzyme constructs were co-transformed in *E. coli* BL21 DE3 Star competent cells (Berkeley QB3 MacroLab; Berkeley, CA, USA) with either the cGAMP biosensor pET31b-Gm0970-P1-4delA-Spinach or the c-di-GMP biosensor pET31b-Dp-Spinach2; kanamycin and ampicillin resistance was used to ensure colonies contained both plasmids. Three biological replicates were picked and grown in 16 mL culture tubes with 2 mL of non-inducing minimal media containing 1 mM MgSO_4_, 0.5% glucose, 1× NPS, 100 μg/mL kanamycin and 50 μg/uL carbenicillin, at 250 rpms and 37°C for 7–8 h. Afterwards, a 1:100 ratio of minimal media cells was transferred into 16 mL culture tubes with 2 mL of auto-inducing media containing 1% tryptone, 0.5% yeast, 1 mM MgSO4, 0.5% glycerol, 0.05% glucose, 0.2% α-lactose, 1× NPS, 100 μg/mL kanamycin and 50 μg/uL carbenicillin, and grown at 250 rpms and 37 °C for 16 h. For in vivo flow cytometry, a 1:100 ratio of auto-inducing media cells was incubated in 100 μL 1× PBS and 50 μM DFHBI-1T for 10 min at room temperature. Each sample was analyzed via flow cytometry (30,000 events). Raw data were processed with FlowJo software (Becton, Dickinson and Company, Franklin Lakes, NJ, USA).

## 4. Conclusions

The RNAseq analysis results demonstrate that affinity capture of riboswitches from the isolated transcriptome, at least under non-covalent conditions, proved to be challenging. In retrospect, the modest enrichment observed in vitro for the cGAMP riboswitch was unlikely to overcome non-specific base-pairing between cGAMP and RNAs, which led to high general background due to non-specific retention of transcripts. We had expected stronger enrichment due to the high affinity previously measured for the RNA–ligand interaction (~530 pM) [8]. However, there are two major factors that would decrease affinity. First, ligand conjugation to Sepharose affects binding to some extent, and the results of our in vitro experiments reflect this issue. The *x*-ray crystal structure of a related riboswitch aptamer bound to cGAMP shows that the 2′ hydroxyl and phosphate groups of the cyclic dinucleotide are solvent accessible, whereas both nucleobases are recognized through H-bonding interactions and buried inside the binding pocket [12]. Since the conjugation chemistry is non-specific, we expect some fraction of cGAMP was conjugated to Sepharose by the backbone and another fraction conjugated by the adenosine. Second, the context of the full mRNA is very likely to affect in vitro re-folding of riboswitches. This was not tested in our in vitro experiments but was apparent in the transcriptome analysis.

Besides the enrichment issues, non-specific base-pairing is particularly problematic for studying the interactions of nucleotide signals with nucleic acids (DNA, RNA) rather than proteins. The RNAseq analysis reflects this issue, as we observed significant reads across the transcriptome retained on the cGAMP beads, whereas the control beads were relatively clean. We expect that a more successful experiment in the future would be to perform a covalent affinity capture reaction, preferably in live cells prior to isolation of RNAs.

Taken together, the reporter results for the GSU1937 genetic deletion clearly show that it affects cGAMP production in the native bacterium, *G. sulfurreducens*, especially when extracellular electron acceptors such as Fe(III) are present. The biosensor results for the fusions strongly support GSU1937 being an active Hypr enzyme that makes cGAMP. While GacA and GSU1937 F4 both show some c-di-GMP synthase activity in *E. coli*, we previously determined that GacA acts selectively as a cGAMP synthase when in *G. sulfurreducens* [15] and it was outside the scope of the current study to address this for GSU1937. Regardless of which cyclic dinucleotide is made, these results are significant because, to our knowledge, it is the first demonstration that an enzyme with a modified GGDDF motif has catalytic activity in vivo. 

The cGAMP riboswitch reporter data not only identified a new enzyme in *G. sulfurreducens* able to produce cGAMP, but showed it is most active in the presence of Fe(III) as an electron acceptor. This, combined with the new transposon mutants showing cGAMP production can be affected by systems targeted to the cell exterior, continues to point to cGAMP having a role in differentiating between types of insoluble electron acceptors. Pilus retraction and surface sensing affects production of cAMP and c-di-GMP of *Pseudomonas* and *Caulobacter* [35,36], but in such model biofilm systems, the primary goal of the bacterium is to attach to a surface. For *Geobacter*, a surface could be inert, or it could offer the opportunity to produce energy. Further complicating decision making for *Geobacter* is the fact that metal particles only support electron transfer for a short duration, while syntrophic partnerships and electrodes can sustain long-term growth. This need to sense not only extracellular materials, but also their value, could drive the evolution of cGAMP signaling networks in bacteria such as *Geobacter*.

## Figures and Tables

**Table 1:** Genes identified by transposon mutagenesis screen.

Strain or Transposon ID	Relative Luminescence ^1^	Disrupted Gene, Transposon Location and Description
WT	100 ± 27.9	
Δ*gacA*	26.6 ± 6.5	GSU1658; Hypr GGDEF
Δ*esnD*	154.1 ± 32.8	GSU3376; Diguanylate cyclase
empty	1.3 ± 1.3	
6G9	27.3 ± 11.6	GSU1508; *xapK*; 463/1119 nt
3F4	35.7 ± 4.1	GSU1508; *xapK;* 730/1119 nt
2G8	41.7 ± 15	GSU1512; Hypothetical
2F9	46.2 ± 44.4	GSU1497; *pilA-C*
1C4	56.4 ± 11.3	11 bp deletion in GSU0946 (Sensor Diguanylate cyclase/Phosphodiesterase EAL, HAMP-; PAS-containing *also* Intergenic insertion, negative strand; GSU0538 (*hspA-1* ATP dependent chaperone)—GSU0539 (hypothetical).
4F11	189.5 ± 107.6	GSU1491: *pilB;* 424/1707 nt
9C1	204.1 ± 52.5	GSU2220; esnB; CheW-like protein
4E6	266.5 ± 41.4	GSU1491; *pilB*; 1522/1707 nt

^1^ Relative luminescence calculated as Relative Light Units per OD^600^, compared to WT, to account for any differences in final cell density.

**Table 2:** Percent binding pocket similarity of *G. sulfurreducens* Cache domain genes to known Cache-ligand structures.

Gene Name	Catalytic Domain	Known Cache-Ligand Structures					
-	-	PctA: AA	Mlp24: AA	Tlp3: AA	CitA:	DcuS:	TlpB:
					Citrate	Malate	Pyruvate
GSU0400	MCP	0.17	0.18	0.22	0.26	0.29	0.28
GUS0962	HisK	0.25	0.28	0.24	0.23	0.22	0.21
GSU1030	MCP	0.6	0.63	0.79	0.82	0.92	0.91
GSU1033	MCP	0.61	0.64	0.82	0.84	0.85	0.84
GSU1303	MCP	0.62	0.66	0.80	0.81	0.90	0.89
GSU2297	HisK	0.5	0.52	0.54	0.56	0.57	0.57
GSU2388	HisK	0.7	0.79	0.99	0.99	0.89	0.89
GSU2507	HisK	0.73	0.79	0.94	0.98	0.91	0.92
GSU3356	GGDEF	0.76	0.8	0.99	0.98	0.89	0.97

DeeplyTough algorithm reports percent dissimilarity scores; shown are 1-dissimilarity scores.

## Data Availability

RNA-seq data will be deposited in NCBI BioProject for *Geobacter sulfurreducens* MN1 strain (accession number PRJNA290373).

## Supplementary Materials

The following supporting information can be downloaded at: https://www.mdpi.com/article/10.3390/ijms23031183/s1.
