# Supplementary material for: The Signaling Pathway That cGAMP Riboswitches Found: Analysis and Application of Riboswitches to Study cGAMP Signaling in Geobacter sulfurreducens
Source: Int J Mol Sci. 2022 Jan 21;23(3):1183. doi: 10.3390/ijms23031183 (PMC8835794; doi:10.3390/ijms23031183)
Supplement: Supplementary file 1 [file ijms-23-01183-s001.zip › ijms-1525455-supplementary.pdf]

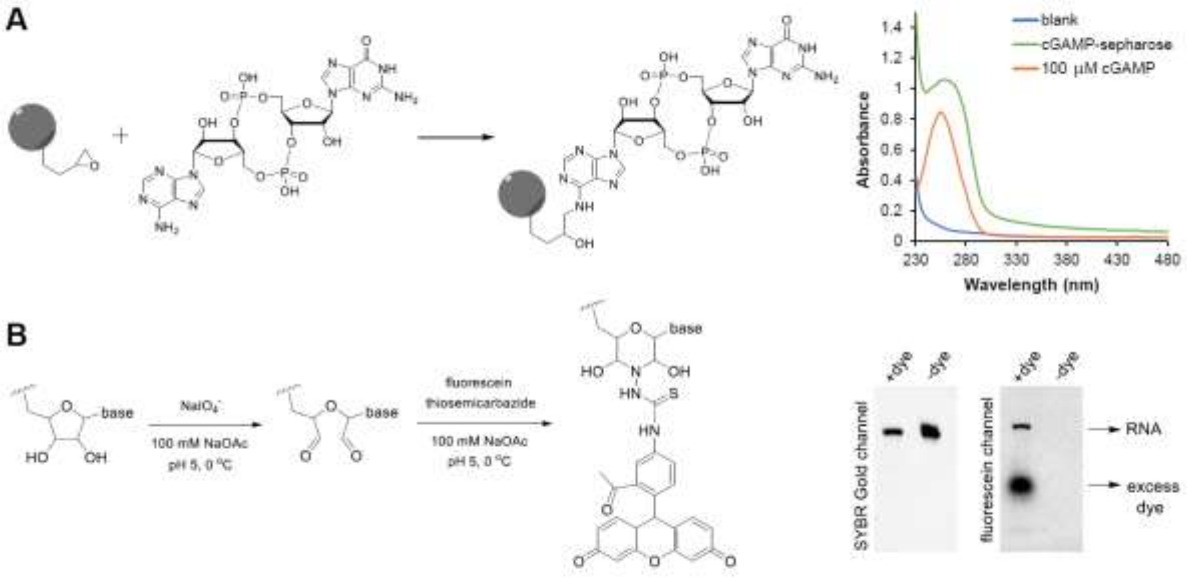

Figure S1. Preparation of (A) cGAMP-Sepharose and (B) 3'-end FITC-labeled RNA for analysis of *in vitro* affinity capture method. Absorbance at 254 nm shows successful conjugation of cGAMP to Sepharose beads. FITC-labeled Gm790 RNA is analyzed by SYBR Gold staining and fluorescence in the FITC channel.

Table S1. Differential expression analysis of riboswitch genes

	input		cGAMP vs input			cGAMP vs blank			blank vs input		
**Gene Name**	baseMean	rank out of 3647	log2Fold Change	pvalue	padj	log2Fold Change	pvalue	padj	log2Fold Change	pvalue	padj
**GSU0183**	509.5711	2602	0.33	0.07	0.42	-0.22	0.32	0.81	0.55	0.01	0.29
**GSU1001**	1355.191	1918	0.44	0.01	0.17	-0.01	0.96	0.99	0.44	0.02	0.34
**GSU1018**	2358.198	1432	0.26	0.21	0.62	0.20	0.37	0.83	0.06	0.77	0.99
**GSU1060**	428.8915	2679	0.30	0.41	0.76	-0.29	0.45	0.86	0.60	0.13	0.73
**GSU1247**	5389.679	666	-0.29	0.27	0.66	-0.30	0.25	0.78	0.01	0.96	1.00
**GSU1556**	1727.897	1711	0.10	0.62	0.88	0.33	0.12	0.67	-0.24	0.27	0.85
**GSU1761**	20418.28	132	0.35	0.13	0.53	0.21	0.35	0.82	0.13	0.57	0.98
**GSU1945**	15516.26	182	0.52	0.10	0.47	0.16	0.62	0.90	0.37	0.25	0.84
**GSU1948**	5259.141	686	0.00	1.00	1.00	0.16	0.43	0.85	-0.16	0.43	0.94
**GSU2033**	825.0176	2298	-0.88	0.25	0.64	-1.20	0.12	0.67	0.32	0.68	0.99
**GSU2504**	653.3774	2454	0.68	0.15	0.55	0.26	0.60	0.89	0.42	0.39	0.93
**GSU2515**	3738.183	973	0.70	0.02	0.23	0.34	0.26	0.78	0.36	0.23	0.82
**GSU2885**	7347.108	481	0.31	0.09	0.45	-0.06	0.75	0.93	0.37	0.05	0.49

For each contrast, log2FoldChange describes the difference in expression of two samples (cGAMP vs input, cGAMP vs blank, blank vs input). Positive numbers indicate that first group is more highly expressed than the second, while negative numbers indicates that the second group is more highly expressed that the first. P-values are calculated with a Wald test and corrected for multiple testing (padj) with the Benjamini and Hochberg method as described in the DESeq2 standard workflow.[21]
